# Dynamics of the lactose content and other osmotic agents in milk throughout lactation according to the cow's parity

**DOI:** 10.3168/jdsc.2024-0717

**Published:** 2025-03-18

**Authors:** A. Hamon, J. Guinard-Flament, A. Costa, A. Fischer, P. Faverdin, M. Gelé, A. Boudon, S. Lemosquet

**Affiliations:** 1 Institut Agro Rennes–Angers, 35042 Rennes, France; 2 Department of Veterinary Medical Sciences, University of Bologna, 40064 Ozzano dell'Emilia (BO), Italy; 3 Institut de l'Elevage, 49105 Angers, France; 4 PEGASE, INRAE, Institut Agro, 35590, Saint-Gilles, France

## Abstract

Milk lactose content (LC) is known to vary due to metabolic disorders and udder inflammation. To assess its ability to serve as a marker of these 2 disorders, it is important to first understand its dynamics for healthy cows, particularly its relation with milk osmolarity, because it is the main osmotic agent. To assess the dynamics of LC, we studied relations among contents of lactose and other major osmotic agents in milk throughout lactation in different parities. Cows were fed a constant TMR diet throughout lactation. The profile of osmotic agents in morning milk (lactose, K, Na, Cl, and protein) was measured in the milk of 30 cows (17 primiparous and 13 multiparous). To avoid confounding effects due to udder inflammation, data from cows with unhealthy udders (i.e., 2 or more milk samples with SCC >200,000 cells/mL) were excluded (n = 2 primiparous and 3 multiparous). From 12 to 235 DIM, 8 to 9 samples per cow were first taken 2 wk apart, and then taken monthly. A regression model that included cow parity, DIM, their interaction, and a random cow effect was used to illustrate the dynamics of milk osmolarity and each osmotic agent throughout lactation. Milk osmolarity remained stable throughout lactation (279 ± 0.3 mOsm/L), with no significant difference between primiparous and multiparous cows. The contents of lactose, K, Na, Cl, and protein explained 86.4% of milk osmolarity. Milk LC was lower for multiparous compared with primiparous cows over the entire lactation period, except at 12 DIM, when both parity cows had similar LC. For multiparous cows, the lower LC was compensated by higher Na content. Principal component analysis of all osmotic agents explained 82% and 83% of the cumulative variance throughout lactation for primiparous and multiparous cows, respectively. At 12 DIM, multiparous cows had lower LC and Cl and K contents compared with primiparous cows, which exhibited only lower LC compared with the rest of the lactation period. From 40 to 235 DIM, however, milk from the 2 parities had similar dynamics of osmotic agents: K content decreased and protein content increased. This study revealed that in cows with healthy udders, the dynamics of LC and other osmotic agents contents in milk differed between parities and throughout lactation to maintain a constant milk osmolarity.

## Highlights

• Milk osmolarity did not change significantly during lactation.
• Milk osmolarity did not differ between primiparous and multiparous cows.
• The lower lactose content for multiparous cows was compensated for mainly by Na.
• The major osmotic agents (lactose, Na, K, Cl, protein) varied throughout lactation.
• Primiparous and multiparous cows had the same osmotic agent dynamics after 40 DIM.

## Graphical Abstract

**Figure:**
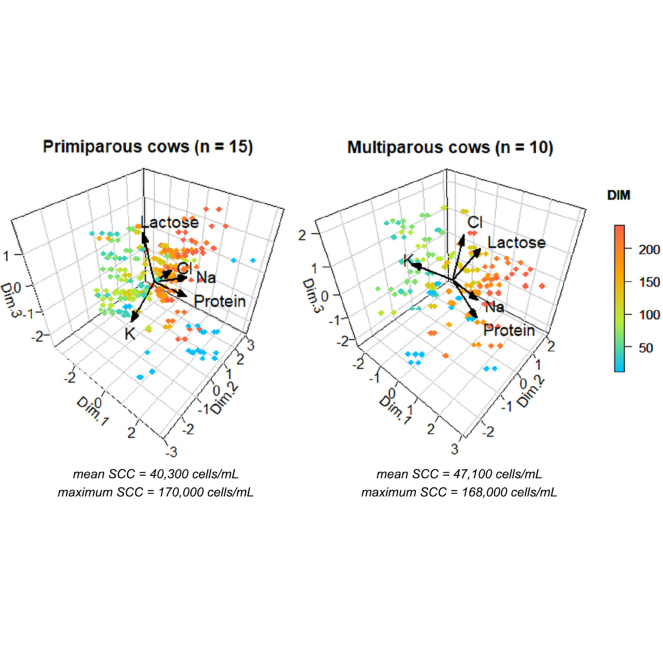
**Summary:** Relations were studied among the major osmotic agents (lactose, Na, K, Cl, and protein) in milk from dairy cows with somatic cell count (SCC) below 200,000 cells/mL of milk and fed a diet with a constant composition. The results revealed that the lactose content and the primary osmotic agent differed between primiparous and multiparous cows and varied mainly at the onset of lactation for each, whereas milk osmolarity did not change. Other osmotic agents varied throughout lactation, and some differed between parities. At the onset of lactation, a low lactose content was associated with lower Cl and K contents for multiparous cows but not for primiparous cows. During the rest of lactation, the profile of osmotic agents followed similar dynamics over the days in milk (DIM) for both parities.

Although the milk lactose content (**LC**) is known to vary little during lactation, it has been the subject of research due to its association with metabolic disorders (Santschi et al., 2016; Hamon et al., 2024) and udder inflammation (Summer et al., 2009; Hamon et al., 2024). Lactose is the main osmotic agent in milk, and according to Brouwer (1981) and Hanuš et al., (2010), it represents 53.8% of milk osmolarity, whereas potassium (K), chloride (Cl), sodium (Na), citrate, and urea represent only 12.7%, 10.5%, 7.2%, 4.6%, and 1.9%, respectively. As a result, the mammary lactose synthesis and secretion into the alveolar lumen primarily regulate the amount of water transferred into milk and mainly determine the milk volume over the lactation. Milk LC decreases only slightly, if at all, after peak lactation. Milk LC changes most during early lactation, increasing by 0.3 to 0.4 percentage points during the first 40 to 50 DIM (Friggens et al., 2007; Haile-Mariam and Pryce, 2017). These dynamics suggest that the profile of osmotic agents changes to maintain osmolarity between the blood and milk compartments (Linzell, 1972). These changes could also explain why primiparous cows have higher milk LC than multiparous cows (Haile-Mariam and Pryce, 2017; Costa et al., 2020).

Mid-infrared (**MIR**) spectroscopy enables the prediction of several major and minor milk components, including LC, Na, and K, to a certain extent of accuracy. It can be used to monitor individual cows and improve the understanding of dynamics of LC by describing changes in the osmotic profile (i.e., the interaction of the main osmotic agents that contribute to maintain milk osmolarity stable). However, the relative contributions of lactose and the other main osmotic agents (K, Na, Cl, and protein) has been less studied. Several studies described them throughout lactation (Oshima and Fuse, 1977; Peaker, 1977) and observed correlations between LC and the content of K, Cl, or Na. However, feeding conditions and udder health status were not considered or explored, even though they can modify milk LC, Na and K contents, and milk osmolarity (Henno et al., 2008; Summer et al., 2009).

Lactose content has recently demonstrated potential as a marker of important health problems in dairy cows, especially mastitis and metabolic disorders (Costa et al., 2020; Hamon et al., 2024). To determine its ability to serve as a marker, it is essential to understand its dynamics for healthy cows, especially in relation to osmoregulation, which remains relatively understudied. The present study aimed to describe relations among lactose and other osmotic agents throughout lactation according to parity for dairy cows with SCC below 200,000 cells/mL of milk and fed a diet with a constant composition. We hypothesized that milk osmolarity would not change during lactation and would not differ between primiparous and multiparous cows, but that maintaining milk osmolarity would require a different profile of osmotic agents, especially for LC during lactation and between parities.

The experiment was conducted from 2015 to 2016 with Holstein cows at IE PL (Installation Expérimentale Production Laitière), INRAE, Dairy Nutrition and Physiology (Le Rheu, France; https://doi.org/10.15454/yk9q-pf68). The protocol was approved by the Animal Ethics Committee and the French Ministry of Higher Education, Research and Innovation (APAFiS authorization no. 3122-2015112718172611). From 9 to 239 DIM,30 lactating cows were monitored (17 first-parity, 9 second-parity, 2 third-parity, and 2 fourth-parity). Cows were recruited as they calved and were housed in freestalls with free-flow access to individual feeders, water, and lying space. Primiparous and multiparous cows produced a mean of 29.8 ± 4.3 and 38.7 ± 5.2 kg/d of milk (±SD), respectively. A single TMR was distributed ad libitum throughout lactation that contained 63.7% maize silage, 10.3% dehydrated alfalfa, 13.6% energy concentrate (described by Fischer et al., 2018), 11.1% soybean meal, and 1% mineral premix. The diet provided 1.51 ± 0.04 Mcal/kg DM of NEL, 135 ± 6 g/kg DM of CP, and 88 ± 2 g/kg DM of protein digestible in the intestine (the INRAE equivalent to MP supply) according to the INRA (2018) feeding system.

Cows were milked twice per day (0700 h and 1600 h) throughout the experiment, and milk yield was recorded. Overall, milk sampling were collected at morning milking in 9 different time points at 12, 40, 67, 95, 123, 151, 179, 207, and 235 ± 2 DIM. On each sampling time points, samples were analyzed for protein content (**PC**) and LC via MIR analysis (Milkoscan, Foss Electric, Hillerod, Denmark), and SCC was determined for milk from the evening milking. The freezing point was measured using thermistor cryoscopy (CryoStar Automatic, Funke Gerber, Berlin Germany) according to ISO standard 5764:2009 (ISO, 2009) and milk osmolarity was calculated based on Raoult's law (Doucét, 1942): Δθ = Kc × Osm, where Kc is the cryoscopic constant of milk (−1.858°C/kg per mol). For all samples, total Na and K were analyzed via atomic absorption spectrometry after diluting milk with nitric acid (Spectra-AA20 Varian, Les Ulis, France). The Cl was measured using a KONE LAB multiparameter analyzer (Kone Instruments Corporation, Espoo, Finland) and all agents (LC, Na, K, and Cl) were then converted to mOsm/L by dividing them by their molar weight in milk (342.3, 23.0, 39.1, and 35.4 g/mol, respectively). The molar weight of PC was estimated at 29.035 g/mol based on the proportion of each protein in milk (Lapierre et al., 2012) and their molecular weights (Farrell et al., 2004), assuming that 1 mmol/L = 1 mOsm/L. To avoid confounding results caused by udder inflammation, cows with 2 milk samples with SCC >200,000 cells/mL were excluded from analysis (2 primiparous cows and 3 multiparous cows). This threshold is commonly used to identify subclinical cases of mastitis (Dohoo et al., 2011). For the 25 remaining cows, milk samples with SCC greater than 200,000 cells/mL (n = 7) or without SCC information (n = 2) were removed. The final dataset included 216 samples from 15 primiparous and 10 multiparous cows.

All statistical analyses were performed using R software (v. 4.0.4, R Core Team, 2021). Univariate analyses were performed separately for each property to assess dynamics of the contents of LC, Na, K, Cl, and PC during lactation according to parity, using the following linear model (*nlme* package):

*y_ij_* = *β*_0_ + *β*_1_Parity*_ij_* + *β*_2_DIM*_ij_* + *β*_3_Parity*_ij_* × DIM*_ij_* + *u_j_* + *ε_ij_*,

where *y_ij_* is the observed LC, osmolarity, Na, K, or Cl of the *i*th milk sample (i = 216) from the *j*th cow (*j* = 25); *β*_0_ is the mean component content when all predictor values were set to zero or to the reference value (primiparous for parity); *β*_1_ is the effect of parity (primiparous vs. multiparous); *β*_2_ is the effect of lactation time points (12, 40, 67, 95, 123, 151, 179, 207, and 235 DIM); *β*_3_ is the effect of the interaction between parity and time points (DIM); *u_j_* is a random cow effect which account for the presence of multiple samples/repeated observations; and *ε_ij_* is the error term.

For the cow-level effect, a spatial exponential correlation structure was used to capture the stronger correlation between samples collected closer in time. The sample- and cow-level residuals were assumed to follow normal distributions centered on zero and with a constant variance $(\sigma_{ij}^{2}$ and $\sigma_{j}^{2},$ respectively). Homoscedasticity and normality of the residuals were assessed visually using plots and adjustments for multiple comparisons were made using the Tukey-Kramer test. Least squares means were estimated using the *emmeans* package and compared using the *multcomp* package. The significance level was defined as a *P*-value equal to or lower than 0.05, whereas statistical tendencies were considered at 0.05 < *P* ≤ 0.10.

Using the raw data of the final data set, principal component analyses (**PCA**) were performed using the *FactoMineR* package to assess relations among the osmotic contents according to lactation stage for each primiparous and multiparous cows individually, due to a significant interaction of parity and DIM for milk Cl. All milk components were scaled within the cow factor to consider repeated measurements to assess dynamics of osmotic agents throughout lactation without potential bias caused by individual cows. The effect of lactation stage (9 classes of DIM) was assessed as an illustrative factor in the PCA. Mean coordinates of individuals in the same DIM class were calculated in the first 2 PCA dimensions. Only factors with loadings greater than or equal to |0.5| were considered when interpreting results.

Based on the LSM, milk osmolarity did not differ between parities (279 ± 0.3 mOsm/L; *P* = 0.17), and altogether, the measured osmotic agents (LC, Na, K, Cl, and PC) contributed 86.4% of milk osmolarity, regardless of parity (*P* = 0.14). Overall, LC contributed the most (53.5%) to milk osmolarity, followed by K, Cl, Na, and protein (16.0%, 10.3%, 6.2%, and 0.4%, respectively). Components not measured thus contributed the remaining 13.6%. The LSM calculated revealed that LC was significantly higher for primiparous cows than for multiparous cows of approximately 0.15 ± 0.045 percentage points at 40, 67, 123, and 179 DIM (*P* < 0.01; Figure 1), whereas the opposite was observed for Na (−145 ± 32.6 mg/L; *P* < 0.01). Similarly, K was lower for primiparous than for multiparous cows, but only at 67 DIM (−107.6 ± 41.5 mg/L; *P* = 0.02), and tended to be lower at 40 and 95 DIM (*P* = 0.07 and 0.10, respectively). For PC and Cl, the LSM of primiparous and multiparous cows did not differ (3.3% ± 0.54% and 1,043.8 ± 49.7 mg/L, respectively).

**Figure 1:**
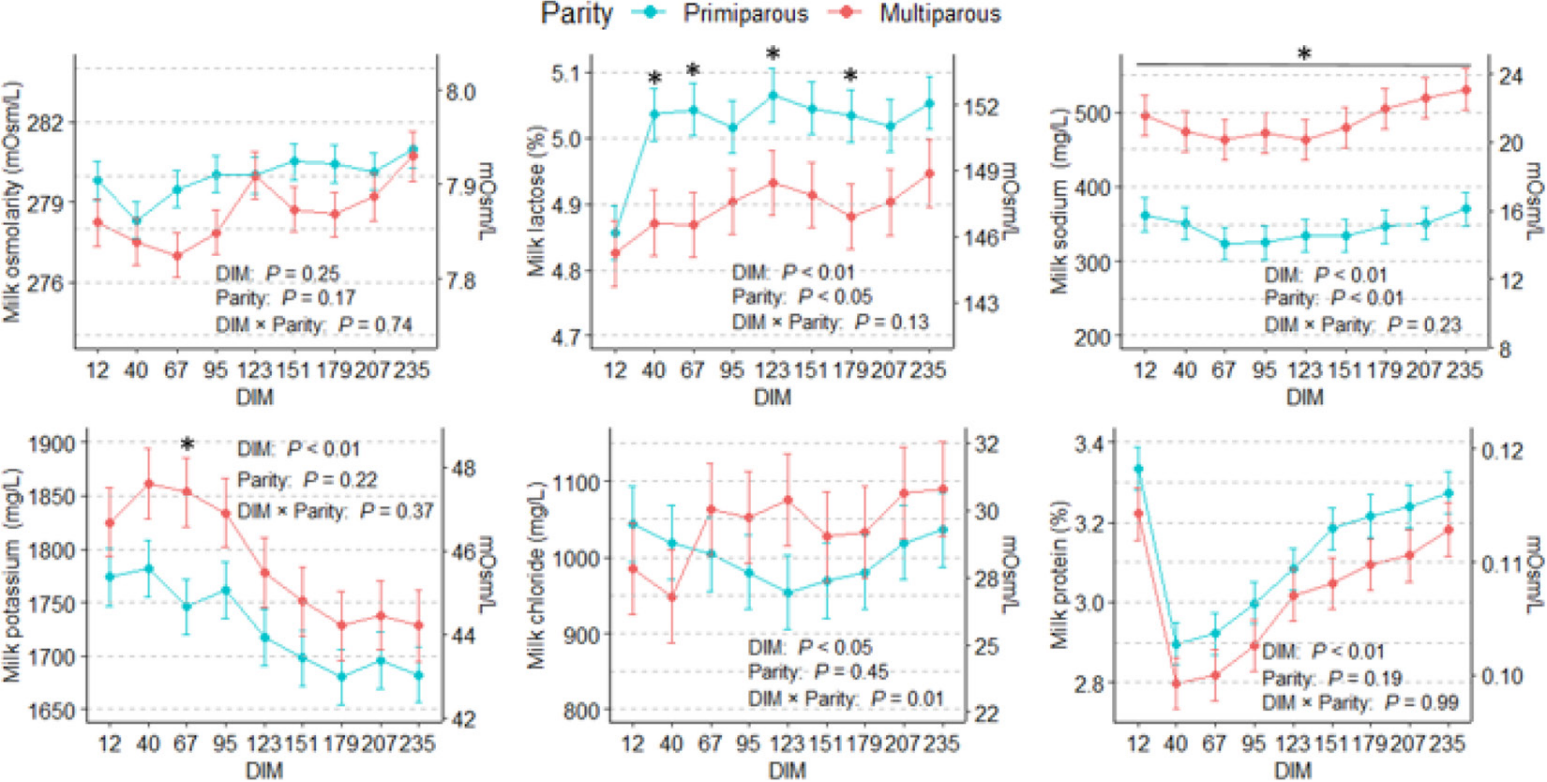
Least squares means of milk osmolarity and milk lactose, sodium, potassium, chloride, and protein contents (mg/L) as a function of the lactation stage (9 time points are different DIM) and parity (15 primiparous and 10 multiparous cows). Asterisks indicate significant (*P* < 0.05) differences between parities at a given DIM. The secondary y-axis displays the contents in mOsm/L. Error bars represent the SE.

Overall, milk osmolarity did not change (*P* = 0.25) throughout lactation. The CV calculated using the raw data was very low (0.92%), which supported the negligible change in osmolarity over DIM. The interaction of parity and DIM was significant for Cl only (*P* < 0.01), but DIM class per se significantly influenced all osmotic agents, which varied throughout lactation (Figure 1). Milk LC was the lowest at 12 DIM for both parities (4.84% ± 0.05%) and then increased rapidly to reach 5.03% ± 0.04% (primiparous) or 4.87% ± 0.05% (multiparous) at 40 DIM. During the rest of lactation, LC and Na remained relatively constant, regardless of the parity. Only one significant difference in milk Na was observed over time (Figure 1; *P* < 0.01). Milk K was higher for both parities in the first 95 DIM than during the rest of the lactation (*P* < 0.01; Figure 1). The LSM suggested nonlinear dynamics for PC, which equaled 3.28% ± 0.04% at 12 DIM, decreased by ∼0.43 ± 0.05 percentage points at 40 DIM, and progressively increased up to 3.23% ± 0.6% at 235 DIM. Regarding the interaction, a significant effect was observed for Cl, as it was stable throughout lactation for primiparous cows (*P* = 0.81), but not for multiparous cows, whose LSM indicate that it is relatively low during the first 40 DIM (967 ± 60.7 mg/L) but then significantly increases from 179 DIM onward (1,083 ± 61.0 mg/L; *P* < 0.05).

The first 3 dimensions of the PCA explained 83% (39.0%, 28.2%, and 15.8%, respectively) and 82% (39.8%, 22.4%, and 19.8%, respectively) of the cumulative variance for primiparous and multiparous cows, respectively (Table 1). For primiparous cows, the loadings opposed lactose (−0.70) to Na (+0.80), Cl (+0.72), and protein (+0.54) in dimension 1, and K (−0.86) to protein (+0.66) in dimension 2. Chlorine contributed to dimension 3 (+0.53). For multiparous cows, the loadings opposed K (−0.72) to Na (+0.81) and protein (+0.83) in dimension 1, whereas LC (+0.95) and Cl (+0.92) contributed to dimensions 2 and 3, respectively. The PCA biplots (Figure 2) revealed that milk samples of the same time point were grouped together chronologically by DIM. Ellipses for samples from intermediate lactation stages overlapped completely or partially, whereas that from early lactation (12 DIM) was isolated (Figure 2). The osmotic agent profile of primiparous cows was related to LC (lower) and Na, Cl, and PC (higher) at 12 DIM. Compared with other DIM classes, this time point had the highest loadings for Na and Cl (both positive), and LC (negative) in dimension 1 (Table 1). At 40 DIM, loadings revealed that osmolarity was described by lower PC and higher K and Cl than those of samples collected at other time points. At 67 and 123 DIM, loadings indicated an osmolarity described by higher LC and K and lower Na, Cl, and PC than those of samples collected at other time points. Conversely, samples collected at 151 and 179 DIM had a different contribution ranking, with lower K and PC. Samples collected from 208 DIM onward were close together and were all characterized by higher Na, Cl, and PC and lower LC and K (Figure 2).

**Table 1:** Coefficients (loadings) of the eigenvectors for each dimension (D.) extracted from principal component analysis of osmotic agents throughout the lactation of primiparous and multiparous cows^1^

Osmotic agent^2^	Primiparous (123 samples)			Multiparous (84 samples)		
	D.1	D.2	D.3	D.1	D.2	D.3
Lactose	−**0.70**	0.34	0.48	−0.01	**0.95**	0.13
Sodium	**0.80**	0.20	0.25	**0.81**	−0.16	0.10
Potassium	0.09	−**0.86**	−0.24	−**0.72**	−0.41	0.25
Chloride	**0.72**	−0.27	**0.53**	0.34	−0.04	**0.92**
Protein	**0.54**	**0.66**	0.41	**0.83**	−0.17	−0.25
Total % explained	39.0	28.2	15.8	39.8	22.4	19.8

1 Values in bold (≥|0.5|) indicate osmotic agents that were strongly associated within the given dimension.

2 Each osmotic agent was scaled within the cow factor to meet the assumptions of independence and normality and to consider repeated measurements.

**Figure 2:**
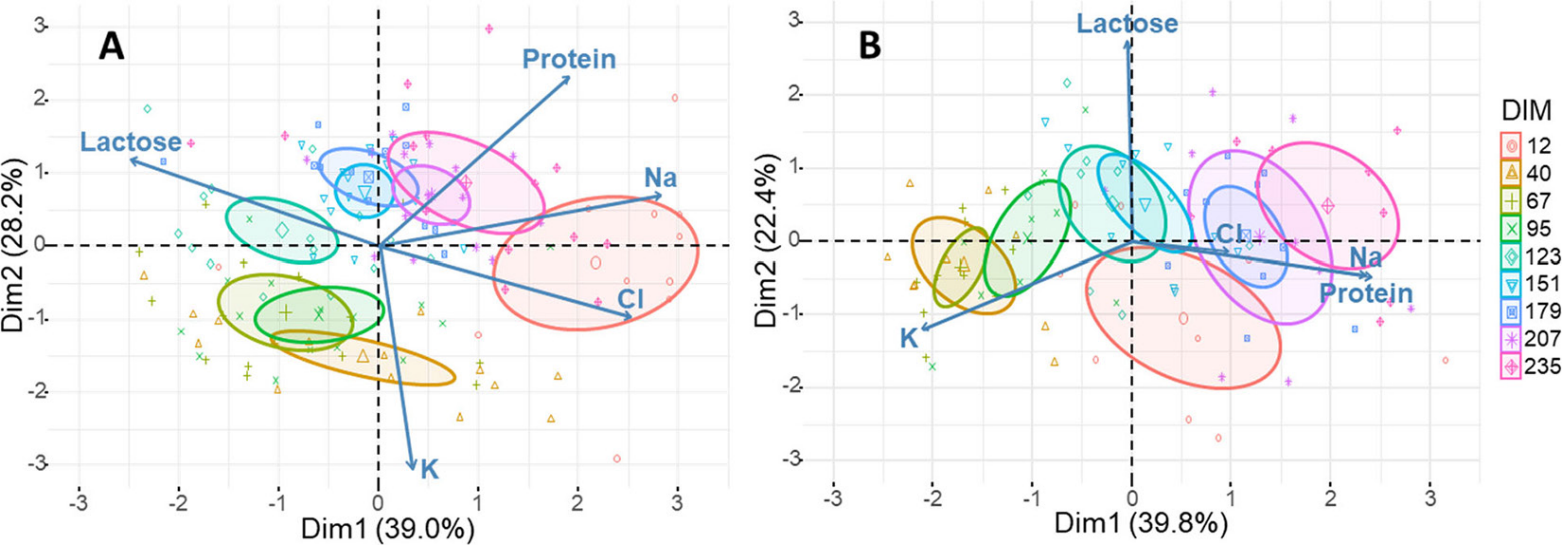
Biplot of the distribution of factor loadings of osmotics agents and samples as a function of the lactation stage (12, 40, 67, 95, 123, 151, 179, 207, and 235 DIM) when milk was sampled for (A) primiparous and (B) multiparous cows. Coordinates of factor loadings were adapted to display individuals and factors in the same space. See Table 1 for factor loadings, which refer only to the factors' absolute positions on the biplots. Dim1 and Dim2 = dimensions 1 and 2.

For multiparous cows, the osmolarity profile at 12 DIM reflected lower LC, K, and Cl, and higher Na and PC than those at the other stages. Milk osmolarity of samples collected at 40 and 95 DIM was described by lower Na and PC and higher K. In primiparous and multiparous cows at 123 and 151 DIM, LC loading was higher. Interestingly, Cl was higher at 67 DIM and lower at 151 DIM. Samples collected in the last 3 time points belonged to overlapping ellipses with lower K and higher Na and PC.

This study examined relations between LC and other osmotic agents to describe changes in the osmotic profile that occur due to physiological changes in LC related to parity and the stage of lactation. The results confirmed that milk osmolarity does not change during lactation or differ between primiparous and multiparous cows. Hanuš et al. (2010) also reported constant osmolarity throughout lactation, with a mean and CV (280 ± 2.3 mOsm/L and 0.8%, respectively) similar to those in the present study (279.8 ± 0.3 mOsm/L and 0.9%, respectively). This consistency is related to the isosmotic gradient between milk and plasma, which entails continuous adjustment of osmotic agents between these 2 compartments (Peaker, 1977). The osmotic agents measured in the present study explained most (86.4%) of the osmolarity. The remaining 13.6% is likely to be the minor osmotic compounds described by Linzell (1972), that is, mainly citrate and urea, together with minerals and ions (e.g., magnesium, phosphate, and calcium), as well as immunoglobulins, antibodies, and enzymes. The mean relative contributions of LC, K, Cl, and Na to milk osmolarity of this study (53.5%, 16.%, 10.3%, and 6.3%, respectively) were similar to those observed by Brouwer (1981) and Hanuš et al. (2010) (53.8%, 12.7%, 10.5%, 7.2%, respectively).

Although parity did not influence milk osmolarity, primiparous cows had higher LC and lower Na content than multiparous cows did throughout lactation, except at 12 DIM. The lower LC in the milk from multiparous cows is generally attributed to compromised epithelial cell integrity, which may result from leaky tight junctions due to different factors including gradual aging of tissue, cumulative damage from repeated clinical or subclinical udder inflammation, and mechanical stress on the epithelium after repeated milkings (Herve et al., 2019; Costa et al., 2025). The lactose leakage causes lactosemia (Peaker, 1977). Consequently, the Na in the alveolar lumen must increase (drawn by the bloodstream) to compensate for the decrease in LC to maintain osmolarity constant. However, leaky tight junctions were not the only reason we observed lower milk LC for multiparous cows. According to Peaker (1977), leaky tight junctions are generally associated with lower LC along with lower K and higher Na and Cl. However, in the present study, milk K and Cl did not differ significantly between multiparous and primiparous cows, but K was numerically higher for multiparous cows (1,789 ± 28.7 mg/L) than primiparous cows (1,726 ± 23.4 mg/L). This difference in milk K between parities may not have been observed due to the study's small dataset and the large SE of the estimates. Two studies observed lower K in milk from multiparous than primiparous cows (i.e., 20–50 mg/L lower; Manuelian et al., 2018; Visentin et al., 2018). These studies considered a longer lactation period (6–450 DIM) and did not restrict samples based on SCC level. Consequently, milk samples with elevated SCC (i.e., up to 12,594,000 cells/mL) were included in the statistical analysis, which could explain the difference with the present study. Supporting this, Visentin et al. (2018) hypothesized that the lower K in milk from multiparous cows was related to the increasing trend in SCC as parity increased and was thus related to changes in tight junction permeability.

The present study explored how the dynamics of LC and other osmotic agents varied throughout lactation to maintain milk osmolarity. Lactose content was lowest for both parities at 12 DIM and increased the most at the onset of lactation. The significant parity × DIM interaction for Cl at 12 DIM indicates that osmotic agents' contribution to milk osmolarity maintenance differed between parities in early lactation. At 12 DIM, primiparous and multiparous cows had similar LC, whereas milk Na was higher for multiparous cows (during the entire lactation) to maintain milk osmolarity although milk Cl was lower (compared with that of primiparous cows). Lactose content remained relatively constant until the end of lactation for both parities, which is consistent with the results of Haile-Mariam and Pryce (2017), but contrasts with those of Friggens et al. (2007), who observed a decrease in LC after the peak of lactation. The dynamics of LC, near-constant or variable after its peak, may be linked to several factors: milk persistency, breed or genetic differences between herds (Haile-Mariam and Pryce, 2017), specific feeding strategies (Friggens et al., 2007; Gulati et al., 2018), or udder inflammation (even subclinical). These studies did not consider udder inflammation status based on SCC, even though it is well known that high SCC is associated with lower LC and a modified milk minerals profile (Summer et al., 2009). Regarding the feeding strategy, we chose a TMR to smooth the peak of lactation and maintain consistent milk production, as indicated by Fischer et al. (2018), which could promote a constant LC.

Although LC remained relatively constant after 40 DIM, the PCA revealed that the osmotic profile varied over DIM, albeit with some similarities between primiparous and multiparous cows. In particular, the sample ellipses were arranged as a function of DIM, and milk K was always negatively correlated with PC in both parities. Changes in the milk osmotic profile appeared to be mainly related to PC and K. In fact, K decreased throughout lactation, whereas PC increased progressively with DIM, which agrees with the results of previous studies (Pogorzelska et al., 2018; Stocco et al., 2019). In terms of osmotic contribution, the variation in milk K between 40 and 235 DIM (3.0 mOsm/L) was larger compared with the variation for PC (0.14 mOsm/L). This negative correlation between K and PC may have been due to a mechanism not related to milk osmoregulation. It may result from decreases in milk yield or udder metabolism during lactation, with a change of energy partitioning from Na-K ATPase pump activity to milk-protein synthesis and turnover (Baldwin et al., 1985; Hanigan, 2005).

In conclusion, this study demonstrated that even though milk osmolarity remains constant, the osmotic agents concentrations in milk vary, as they compensate for each other to maintain the consistency of the osmotic gradient between milk and plasma. A key strength of this study was the availability of punctual concentration for the 4 main osmotic agent: lactose, K, Na, Cl. The osmotic profiles of primiparous and multiparous cows revealed differences in milk LC between the 2 parities that were compensated for mainly by milk Na, but regardless of parity, the findings demonstrated that the osmotic agents' contribution and compensation mechanisms vary within the lactation. Future research should investigate dynamics of lactose and other osmotic agents in cows with metabolic disorders, clinical mastitis, or subclinical udder inflammation to better understand how they vary in response to different health problems and different mastitis-causing pathogens. In perspective, MIR spectroscopy, nowadays used to assess milk gross composition in the field worldwide, could be exploited to predict milk osmolarity at the individual level from milk spectra on a routine basis in the framework of the official test-day analyses. In fact, in addition to the milk LC routinely predicted via MIR, having information about milk osmolarity at cow individual level could help in the identification of cows with subclinical mastitis and, potentially, of the pathogen(s) involved.
